# Importance of Advanced Detection Methodologies from Plant Cells to Human Microsystems Targeting Anticancer Applications

**DOI:** 10.3390/ijms26104691

**Published:** 2025-05-14

**Authors:** Mostafa M. Gouda, Eman R. Elsharkawy, Yong He, Xiaoli Li

**Affiliations:** 1 College of Biosystems Engineering and Food Science, Zhejiang University, Hangzhou 310058, China or goudarowing@yahoo.com (M.M.G.); yhe@zju.edu.cn (Y.H.); 2 Department of Nutrition and Food Science, National Research Centre, Giza 12622, Egypt; 3 Center for Health Research, Northern Border University, Arar 73213, Saudi Arabia

**Keywords:** plant metabolomes, biosensation, evaluation of bioactive molecules, targeted technological therapy, phytochemicals, bioactivity, molecular pathways

## Abstract

The growing global demand for phytochemicals as bioactive sources is prompting scientists to develop methods that link their sensory properties to their mechanisms of action in cancer treatment. Recent techniques for tracking the actions of small plant metabolites (SPMs) from single-cell plant sources to their molecular anticancer biomarkers could provide valuable insights in this field. Among the critical methods discussed in this review are the real-time tracking of cell components through stable isotope probing (Sis) and microspectroscopy, which has attracted the attention of biotechnologists. Additionally, the precise pathways required for studying new insights into functional materials are discussed, based on high-resolution and accurate technologies, which could aid their functional categorization. Notably, the molecules under study have recently garnered attention for their anticancer applications due to advancements in effective evaluation techniques that surpass traditional methods. In December 2020, the Food and Drug Administration (FDA) authorized 89 SPMs as safe anticancer natural molecules. In conclusion, by combining spatiotemporal techniques and SPMs’ mechanisms, they could facilitate the development of more exceptional, bio-efficient materials.

## 1. Introduction

In recent years, the growing demand for bioactive compounds derived from plants has led to a huge shift in cancer treatment, as researchers explore more targeted and less toxic therapies. Among these compounds, SPMs have gained significant attention due to their low toxicity and selective ability to target cancer cells, offering a promising alternative to conventional chemotherapy, which often results in severe side effects due to their inability to differentiate between healthy and cancerous cells [1,2]. Unlike traditional broad-spectrum cytotoxic agents, targeted therapies, particularly those involving small molecules, provide a more refined approach, maximizing therapeutic efficacy while minimizing harm to healthy tissues [3,4,5,6,7]. These compounds, including terpenoids, flavonoids, phenolics, and alkaloids, are increasingly recognized for their diverse and potent selective anticancer properties [8,9].

The bioactivities of these molecules are primarily attributed to their ability to interact with cellular pathways, promoting selective targeting of cancer cells. For instance, compounds such as curcumin, found in turmeric, and gallic acid, present in various fruits, have been shown to modulate critical signaling pathways, including PI3K/Akt and NF-κB, contributing to the inhibition of cancer cell proliferation, induction of apoptosis, and prevention of metastasis [10,11,12,13]. Moreover, these phytochemicals have been found to exhibit synergistic effects when used in combination, enhancing their therapeutic potential [2,14]. The molecular mechanisms underlying these effects, as well as their ability to modulate multiple biological processes within cancer cells, underscore the therapeutic promise of SPMs.

More importantly, advances in molecular technologies for plant and cancer cell detection, such as real-time metabolic monitoring and high-resolution tracking of cellular processes, further support the shift toward this approach. Techniques such as stable isotope probing (Sis) and Raman microspectroscopy allow researchers to track the metabolic pathways of SPMs in living plant cells, providing a deeper understanding of their mechanisms for releasing SPMs [14,15,16]. These technologies offer insights into the molecular interactions between SPMs and cancer cells and help elucidate the complex metabolic networks crucial for cancer progression. By combining these innovative technologies with advanced spectroscopic techniques, scientists can now visualize and quantify the impact of SPMs on cellular metabolism with unprecedented precision, paving the way for more effective cancer therapies [17,18,19,20,21].

This growing body of knowledge enhances our understanding of genomics and facilitates practical advancements in biotechnology [8,22]. In addition, preliminary genome analysis has revealed the presence of specific genes related to lipid biosynthesis and stress response in cancer cells treated with monoterpene (e.g., thymol; THY), making them vital biosensation cell models for investigating the functional roles of these molecules [2,18,23]. The integrated spectroscopic-stable isotope approach offers a promising method for comprehensive genome and metabolome analyses, providing valuable insights into molecular composition, metabolic activity, and transcriptomic structure.

Therefore, this review aims to synthesize current knowledge on the role of SPMs in cancer treatment, emphasizing the cutting-edge technologies used to track their bioactivity and the molecular pathways they influence. By exploring these bioactive compounds’ chemical structures, target pathways, and mechanisms of action, we aim to highlight their potential key roles in developing more targeted and efficient anticancer therapies. The integration of novel technologies and the growing body of research into SPMs’ anticancer properties promise to open new avenues for cancer treatment and may lead to the development of more effective, personalized therapeutic strategies in the near future.

## 2. Definition of Small Plant Metabolites (SPMs) and Their Importance

Small plant metabolites are a class of bioactive compounds derived from plants, gaining significant attention due to their therapeutic potential in various diseases, especially cancer [24]. These molecules are generally characterized as low-molecular-weight with antioxidant, anticancer, and anti-inflammatory properties. Their unique chemical structures allow them to interact with cellular pathways, offering selective targeting of diseased cells, including cancerous ones, while minimizing damage to healthy tissues [2,5].

The term small molecule is derived from the number of carbon (C), nitrogen (N), and oxygen (O) atoms that form these simple structures. For example, gallic acid consists of three hydroxyl groups on benzoic acid, which are fewer than the complex structures of polymeric macromolecules like proteins, polysaccharides, or proanthocyanidins, which consist of several smaller subunits of catechins and epigallocatechin [2,25]. Additionally, SPMs should be structurally well-optimized by molecular modeling and analytical methods to facilitate their evolution in serving specific metabolic functions, including regulating endogenous enzyme pathways within cancer cells.

These molecules can be categorized into groups based on their major chemical structures, such as terpenoids, flavonoids, phenolics, alkaloids, and carotenoids, where they are integral to the plant’s defense mechanisms and offer potential health benefits to humans. For example, curcumin, a well-known phenolic compound, modulates key signaling pathways like NF-κB and PI3K/Akt, which are critical in regulating cancer cell proliferation, apoptosis, and migration [26]. Furthermore, several studies have shown that SPMs can influence the activity of enzymes involved in tumor growth and metastasis, making them viable candidates for cancer therapy [2].

The growing research on SPMs highlights their importance as therapeutic agents and key players in developing functional foods and nutraceuticals. As the field progresses, integrating SPMs into clinical applications, especially in cancer treatment, holds great promise. However, bioavailability and delivery methods must be addressed to fully realize their therapeutic potential.

### Importance of a Comprehensive Database of SPM Metabolite Functionalities

It is worth noting that the comprehensive screening database of SPM metabolite functionalities could be a significant solution for applying their diverse configurational structures in food and medical therapy while avoiding the rediscovery of their known functionalities [27]. An extensive evaluation of small-molecule targeted anti-cancer therapies to better support their development should be the target for novel insights in this critical field. In this context, the identified protein targets of approved pharmacological agents may serve as valuable indicators in elucidating the molecular functions of these compounds. Additionally, marketed small-molecule medications and significant therapeutic candidates from clinical studies will be showcased for each target. For example, maytansine inhibitors of enzymes that facilitate the transfer of γ-phosphate groups to protein residues with hydroxyl groups, such as those of protein kinases, could be significant representatives of the SPMs [27]. This group could play essential roles in the differentiation, growth, and proliferation of cancer cells and their modes of action. The human cellular proteome comprises approximately 535 distinct protein kinases, each of which warrants thorough examination and investigation [28]. Additionally, this group activates the caspase-3/7 pathways to induce apoptosis and attaches them to microtubules, initiating mitotic arrest. The protein CKAP5 interacts with DM1 in the trastuzumab emtansine (T-DM1) complex, facilitating the destruction of the cellular membrane and increasing calcium (Ca^2+^) influx. This disrupts the microtubule structure, leading to cytotoxicity and growth inhibition.

On the other hand, integrating molecular detection technologies for categorizing SPMs has improved the spatiotemporal exploration of their action mechanisms in biological cells and tissues through changes in their chemical compositions [2]. Thus, combining phenotypic assays with the molecular mechanisms of action is the optimal and well-established solution for assessing the potential anticancer bioactivity of these molecules. The application of analytical techniques has seen significant advancements, facilitating the discovery and prediction of the precise structural impacts of these molecules.

## 3. Advanced Micro-Spectroscopy Technologies in Single Plant Cell Targeting Its Metabolomics

Recent advancements in detection technologies and Sis have improved our understanding of SPMs’ bioactivities by allowing real-time tracking of their metabolic pathways in living cells [14,29]. These techniques facilitate the exploration of SPMs’ mechanisms of action at the cellular level, providing insights that could lead to the development of more efficient and bioavailable formulations. The latest advancement in micro-spectroscopy offers high-resolution, non-destructive analysis of microalgae cellular structures, which enhances our understanding of the new generation of microalgae metabolic diversity [17]. These techniques, such as Raman and fluorescence microspectroscopies, provide information about microalgae’s cellular structures, molecular composition, and evolution [14]. They could be used to identify genomic variations, mutations, and structural changes within individual microalgae cells (Table 1).

The differences between micro- and macro-spectroscopy also refer to the spectroscopic technique’s levels that could relate to the Sis mode of action. The micro-level case also provides a detailed, high-resolution analysis of microscopic samples at the sub-micrometer scale, which can efficiently capture microalgae’s cellular structures, molecular composition, and metabolic processes. On the other hand, macro-spectroscopy refers to millimeter- to centimeter-scale spectroscopic measurements encompassing biomasses and bulk materials.

Several discoveries in single-cell microalgae metabolism, made possible through Sis, were connected with microRaman technologies. The study by Ota et al. [30] focused on the fabrication process by which *Euglena gracilis* synthesizes paramylon. This polysaccharide serves as an energy reserve, as demonstrated by Sis-microRaman technology. A lab-on-a-chip microscale technique that sorted and isolated specific *E. gracilis* cells based on their properties enabled the precise tracking of the biological pathways of the microalgae cells. The semiclosed microchannel chips monitored the cells’ metabolic activities and the formation of subcellular granules, specifically polysaccharide paramylon, through Raman microscopy combined with Sis. Indeed, two Sis were prominently utilized to investigate metabolic processes and the biogenesis of paramylon. In that study, the first Sis was deuterium (^2^H) through its incorporation into *E. gracilis* cells by replacing regular water (H_2_O) with ^2^H_2_O.

**Table 1:** Summary of real-time tracking and spatial mapping techniques.

Technique	Description	Applications	Advantages	Reference
**Stable Isotope Probing (Sis)**	It utilizes stable isotopes to track metabolic pathways in real time.	Monitoring the biosynthesis of bioactive compounds and tracking nutrient usage.	High specificity, non-destructive.	[31]
**Raman Microspectroscopy**	Provides molecular fingerprints of cells based on vibrational spectroscopy.	Identify genomic variations and track the accumulation of metabolites.	Label-free, high resolution.	[32]
**NanoSIMS**	High-resolution spatial mapping of stable isotopes in cells and tissues.	Mapping nutrient utilization and visualizing metabolic activity.	High spatial resolution, multi-isotope detection.	[33]
**Hyperspectral SRS**	Combines Raman spectroscopy with hyperspectral imaging for 3D molecular maps.	Visualizing the biosynthesis of metabolites and mapping the distribution of lipids and carotenoids.	High sensitivity, 3D imaging.	[34]
**Integration of Sis and Raman**	Combines Sis and Raman spectroscopy for real-time metabolic tracking.	Tracking paramylon biosynthesis and monitoring metabolic activity in microalgae.	Real-time monitoring, non-destructive.	[14]

Incorporating ^2^H allowed us to trace its presence in metabolic products, particularly in carotenoids, as a critical metabolite of photosynthesis. The study noted that substituting protons in carotenoid molecules with deuterium (^2^H) resulted in observable changes in the Raman spectra, indicating a change in metabolic activity. The second Sis was carbon-13 (^13^C), which replaced the carbon dioxide (CO_2_) in the culture medium with ^13^CO_2_. This substitution facilitated the tracking of carbon incorporation into organic products synthesized by the cells, especially paramylon.

### Important Example for Single Plant Cell Tracking Technology by Raman-Sis Metabolic Mode of Action

**Figure 1:**
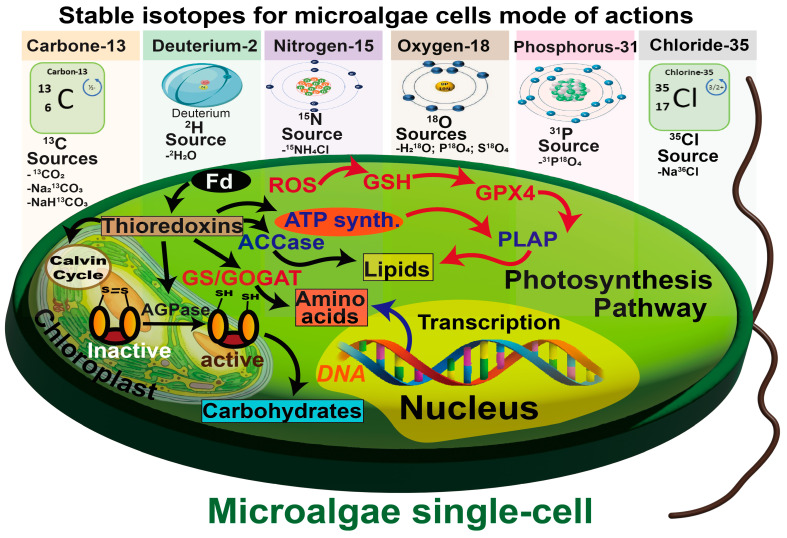
Commonly used stable isotopes in microalgae ecosystem investigations related to carbon fixation and metabolomic implications [32,37].

To track the metabolism of single plant cells, a sophisticated network of interactions among macromolecules and molecules (e.g., lipids, proteins, pigments, and polyphosphates) within individual cells can provide insights into the spatiotemporal dynamics of these metabolites [15,16]. For example, the different metabolic pathways may produce new, innovative functional compounds. Additionally, Mo, Ma, Yan, Cheung, Yang, Yao, and Guo [18] employed untargeted metabolomics to investigate the metabolomic characteristics and detailed toxic mechanisms of erythromycin in *Raphidocelis subcapitata*. In their study, metabolomics analysis of algae revealed that fatty acid biosynthesis and purine metabolism were among the key metabolomic pathways involved in promoting its growth and functionality. Moreover, a metabolic investigation has been extensively engaged in the field of bioengineering for synthesizing intermediates of polyunsaturated fatty acids (especially omega-3) and carotenoids [18,35] (Figure 1).

**Figure 2:**
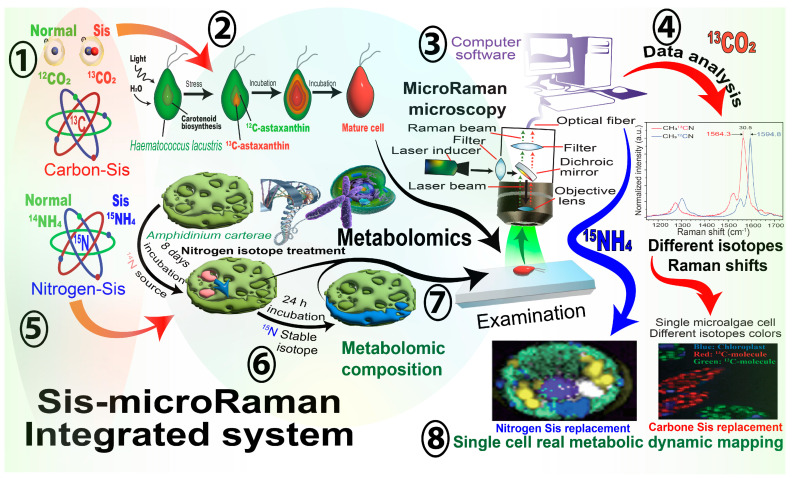
An overview of the method of isotope labeling and microRaman spectroscopy. (1–4) Real-time tracking of *Haematococcus lacustris* single-cell metabolism with ^13^C stable isotope (Sis). (5–8) *Amphidinium carterae* single-cell ^15^N-Sis chemometric visualization by micro-Raman [14] (Copyright permission no: 6013410212131).

Consequently, when substrate atoms are changed with Sis heavier atoms, the vibrational frequency switches to a lower state. As a result, the Raman signal shift will change accordingly, and a new peak shift will appear in a different region [36]. By tracking the changes between the Sis and the same normal atoms during the incubation process, changes in microalgae cell metabolomes can be calculated using models for biomarker molecules, such as DNA, protein, and lipids (Figure 2).

Raman-based Sis studies have mainly focused on the functional characterization of comprehensively labeled microalgae. Commonly used stable isotopic labeling atoms include carbon-13 (^13^C), nitrogen-15 (^15^N), deuterium (^2^H), and oxygen-18 (^18^O) [37] (Figure 2). ^2^H performs best in monitoring single-cell metabolic changes, as it visually minimizes the isotope effect’s impingement [38]. ^13^C and ^15^N can also work as practical tools to decipher multistep and multipoint metabolic pathway by comparing labeled and unlabelled microalgae cell DNA and RNA [32] (Figure 1).

For instance, Fu et al. [39] used ^13^C isotope for monitoring Chlorella microalgae carbon consumption. They concluded that Chlorella had a lower C:N ratio than other microbial species, such as Bacteroidetes. Additionally, Zachleder et al. [31] examined the isotopic effect of deuterium (D_2_O) on the autotrophic growth of algal cells and reported that microalgae cells could resist up to 70% of D_2_O.

## 4. The Potential Application of Spectroscopy and Sis Integration in Plant Studies

**Figure 3:**
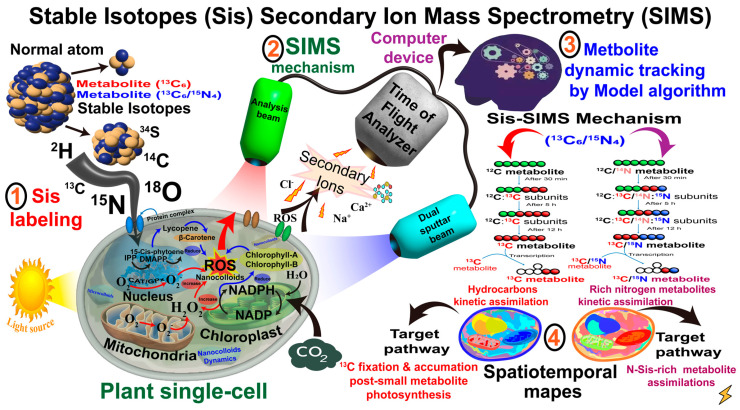
Molecular genomic variation and advances in spectroscopy and microalgae field [46].

The integration between Sis labeling and advanced spectroscopy enables the tracking of plant cells’ nutrient colloidal systems [14,40] (Table 2). For example, secondary ion mass spectrometry (SIMS) and NanoSIMS techniques within nanocolloidal systems enables the spatial mapping of stable isotopes [41,42] (Figure 3). These technologies include stable isotope ratio analysis (SIRA), which offers subcellular resolution imaging capabilities for measuring the stable isotope ratios of specific elements, such as carbon (^13^C, ^14^C), nitrogen (^15^N), deuterium (^2^H), sulfur (^34^S), and oxygen (^18^O) [33]. Additionally, their dynamics and metabolic processes within nanosystems offer detailed insights into plant cellular discoveries [42]. Commonly used isotopically labeled amino acids, such as L-arginine (^13^C_6_), which contains six ^13^C-labeled carbon atoms, are suitable for tracking carbon fluxes in metabolic pathways [43,44]. On the other hand, L-Arginine (^13^C_6_/^15^N_4_), with extra neutrons, provides more comprehensive information on specific amino acid metabolism [45] (Figure 2).

## 5. The Linkage Between Plant Cell Studies and Their Metabolite Applications in Cancer Studies

The novel linkage between plant cell detection technologies and their metabolite applications in cancer leverages the precision and depth of analysis that can uncover new bioactive compounds, optimize their production, and develop innovative cancer therapies [23,47]. This interdisciplinary approach can potentially transform both plant science and oncology, paving the way for groundbreaking discoveries in the fight against cancer. This approach focuses on discovering new drugs and emphasizes understanding the molecular mechanisms underlying their effects [48]. For instance, a particular metabolite that targets a specific protein or pathway involved in cancer progression can be predicted from primary plant sources using models that employ artificial intelligence (AI) and machine learning with spectroscopic data analysis, which holds immense promise [49,50]. These models can process vast amounts of spectral data to identify patterns and predict the biological activity of unknown metabolites, accelerating the discovery of new anticancer agents. By understanding the metabolic pathways involved in producing anticancer compounds from various sources, researchers can utilize genetic or metabolic engineering to enhance their yield in plant cell cultures, potentially leading to other effects for the same compound obtained from different plant sources [51]. For instance, spectroscopic technologies can help uncover new compounds with similar or even more potent effects. A metabolite-identified model developed through Raman spectroscopy may exhibit vigorous activity against cancer cell lines in vitro, while modeling them from their plant sources and mechanisms of action. Such a linkage approach could lead to groundbreaking discoveries in the oncology field and studies on animal signaling pathways involved in cancer progression. This provides detailed insights into the chemical composition and metabolic processes that could aid in modeling discoveries in SPM molecules, such as alkaloids, flavonoids, and terpenoids, within plant cells without damaging them. This is particularly useful for identifying regions of high metabolite concentrations, which can be targeted for further anticancer studies.

## 6. The Core Biological Applications of Detection Technologies in Cancer Cells Are Different Approaches from the SPM Metabolites’ Mode of Actions

**Figure 4:**
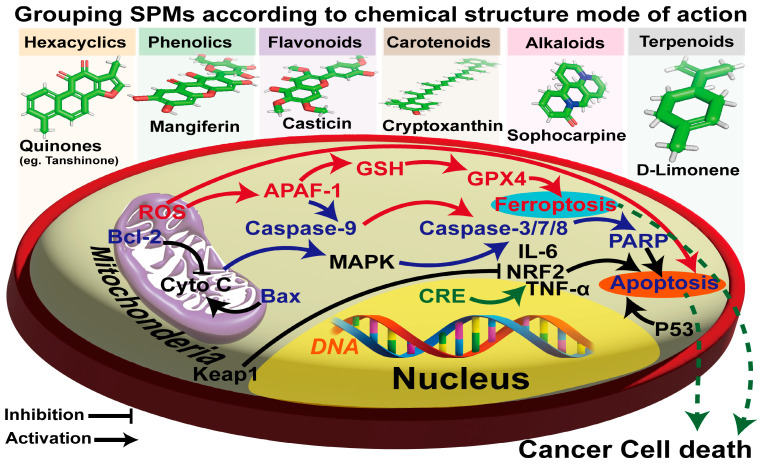
The connected action mechanisms for grouping SPMs according to their potential functionalities in the cancer cells [53].

The variable biological activities of SPMs against cancer lie in their diverse chemical classes, which are characterized by complex molecular structures [52]. Furthermore, the diverse chemical classifications contribute to the enhanced antioxidative and pro-oxidative activity exhibited by these dietary phytochemicals. These compounds can be divided into significant classes: hexacyclic (e.g., quinones), phenolics, carotenoids, alkaloids, and terpenoids. As a notable example, terpenoid groups have recently demonstrated a high degree of linkage among the modes of function of their active groups in specific cancer pathways, as illustrated in Figure 4 and Table 3.

For instance, Kim et al. [54] reported that gallic acid, a plant phenolic compound with three hydroxyl groups on a benzoic acid backbone, exhibits significant cytotoxic effects on cancer cells. Recent studies have highlighted gallic acid’s role in inhibiting cancer cell proliferation, invasion, and metastasis, offering vital insights into its therapeutic potential in oncology settings. Tang et al. [55] highlighted the ability of gallic acid to reduce the viability of HeLa cancer cell lines, a phenomenon mediated by the upregulation of AMPK pathway gene expression due to its interaction with the AKT gene.

**Table 3:** The importance of classified phytochemicals in targeting specific pathways.

Phytochemical Class	Key Compounds	Mechanisms of Action	Pathways Affected	Reference
**Terpenoids**	D-limonene, Cucurbitacin	Induces apoptosis, inhibits the PI3K/Akt pathway, and disrupts the cell cycle.	PI3K/Akt, JAK2/STAT3	[56]
**Phenolics**	Curcumin, Gallic acid	Modulates NF-κB, induces cell cycle arrest, and promotes apoptosis.	NF-κB, Cyclin D1/CDK4	[57]
**Flavonoids**	Quercetin, Catechins	Inhibits Wnt/β-catenin, induces oxidative stress, and promotes apoptosis.	Wnt/β-catenin, ROS	[58]
**Carotenoids**	β-carotene, β-cryptoxanthin	Modulates oxidative stress, inhibits EMT, and induces apoptosis.	TGF-β1, MMPs	[59]
**Alkaloids**	Sophocarpine, Vinblastine	Induces apoptosis, inhibits inflammation, and reduces cytokine production.	Caspase-3, TNF-α, IL-6	[60]

Furthermore, in vitro investigations have revealed a substantial reduction in IL-6 levels in PC3 cells following treatment with gallic acid at varying concentrations. This reduction subsequently leads to the downregulation of p38, JNK, PKC, and PI3K/Akt signaling pathways, ultimately impeding cell invasion and proliferation. Another important family is quinones, a class of organic compounds containing either a cyclohexadiene or dimethylene structure. This family has several bioactive compounds (SPMs), such as anthraquinones, in various microalgae types, including *Chlorella sorokiniana*. While the mechanism of anthraquinone in treating liver cancer is complex, research has indicated that it primarily plays an anti-cancer role through the generation of reactive oxygen species (ROS), induction of apoptosis, and DNA damage repair [61].

The pharmacological relevance of plant phytochemicals suggests their potential as medicinal agents for treating a wide range of human cancers and malignant tumors. Wilson et al. [62] mentioned the important role of phytochemicals, as functional foods, in oxidative disease prevention. They overviewed the role of antioxidants in inhibiting the acetylcholinesterase enzyme, which is one of the risk factors for cancer-associated diseases. For instance, taxol (known as paclitaxel) is a common anticancer agent consisting of a diterpene taxane ring, which can be obtained from the Taxus brevifolia tree plant [63]. This diterpene compound induces cytotoxic activity by increasing the polymerization of tubulin, thereby disrupting the dynamics of tubulin–microtubule interactions. The confirmed antagonistic effects of taxol have been reported in metastatic ovarian, bosom carcinomas, breast cancer, lung cancer, oesophageal adenocarcinoma, and prostate cancer. On the other hand, docetaxel, a more potent relative of the paclitaxel family with higher activity, showed numerous side effects after prolonged exposure, including arrhythmia, neutropenia, and cardiovascular breakdown [64].

## 7. Meta-Analysis of Articles on SPMs as Bioactive Anticancer Agents

**Figure 5:**
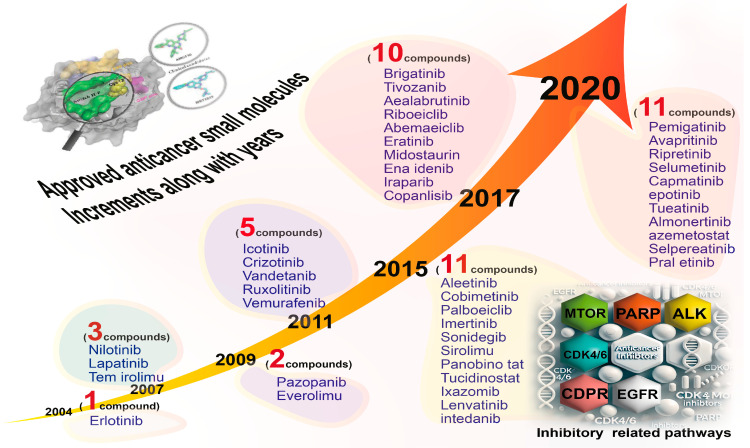
Timeline for approving small-molecule SPM targeted anticancer drugs (Adapted from Zhong et al. [5]; Open access license).

**Figure 6:**
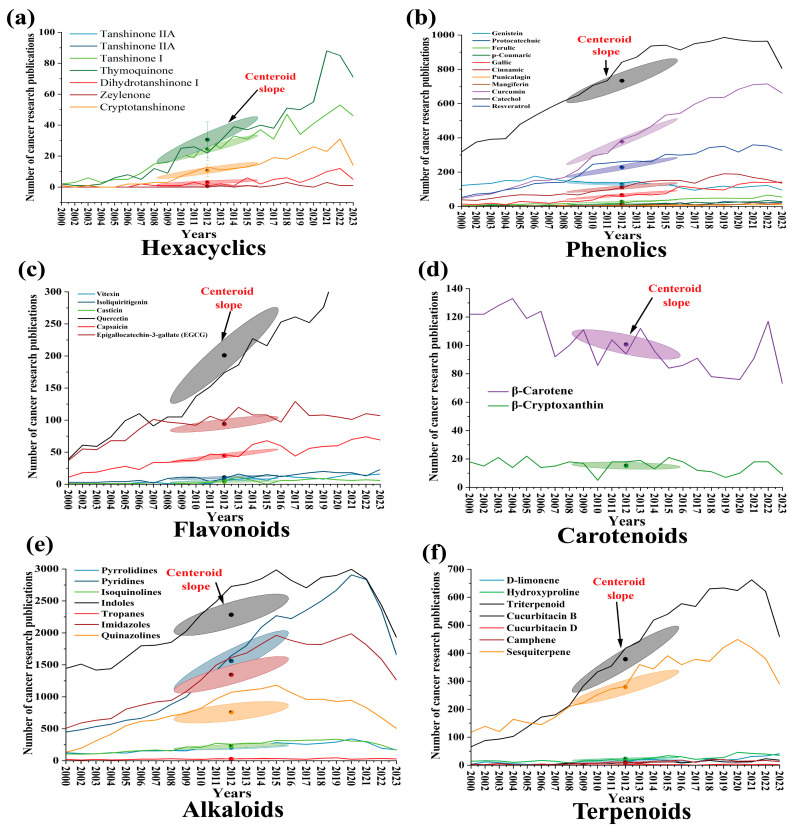
The increments in publications of 50 SPM anticancer agents from 2000 to 2023, including hexacyclic (**a**), phenolics (**b**), flavonoids (**c**), carotenoids (**d**), alkaloids (**e**), and terpenoids (**f**). Data adapted from the PubMed database.

Over the years, the increasing approval and release of small molecules as targeted anticancer agent by different international organizations has led to the urgent need to categorize these small molecules according to their functional impacts. As shown in Figure 5, the number of approved compounds by the US-FDA increased significantly from 2001, beginning with Imatinib, a 2-phenylamino-pyrimidine derivative protein and the first-line therapy recommended for chronic myeloid leukemia (CML) patients in China, to over 11 approvals of small molecules in 2020 for inhibiting specific cancer pathways [5]. In addition, based on the PubMed database (2000–2023), SPM-related publications in cancer research show a noticeable increase in specific groups like alkaloids and phenolics compared to flavonoids and carotenoids (Figure 6).

### Database of Articles on SPMs as Bioactive Anticancer Agents Used for Meta-Analysis

To extract data for the database of articles on SPMs as bioactive anticancer agents, a comprehensive literature search was conducted using the PubMed database https://pubmed.ncbi.nlm.nih.gov/ (accessed on 1 December 2024), spanning publications from 2000 to 2023 (Figure 6). The search focused on identifying studies that reported the anticancer activity of SPMs and their underlying mechanisms. The search query included a combination of keywords such as “bioactive compounds”, “anticancer agents”, “phytochemicals”, “terpenoids”, “flavonoids”, “phenolics”, and “cancer pathways”. Articles were selected based on their relevance to SPMs’ biological activity, particularly those that linked molecular pathways, chemical structures, and their effectiveness in preclinical or clinical cancer studies. After initial screening, articles focused on experimental data, clinical trials, or meta-analyses of SPMs’ anticancer potential were included. The data extraction process involved compiling information from study titles, abstracts, and full-text articles, specifically noting the type of SPMs studied, targeted cancer types, and the reported outcomes. Only studies that provided sufficient methodological detail on the anticancer effects of SPMs were included for analysis, ensuring the integrity and relevance of the database. This systematic extraction from PubMed facilitated the creation of a robust dataset for the meta-analysis, offering insights into the therapeutic potential of various plant-derived metabolites in cancer treatment.

**Figure 7:**
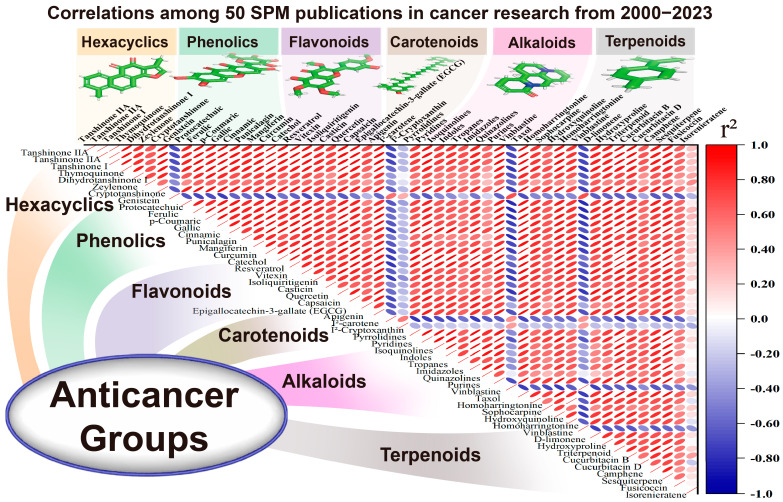
The correlation heatmap among the 50 SPM anticancer agents’ publications was constructed using Origin 2022 (Massachusetts, USA). The data in this figure were adapted from the PubMed database.

A positive significant correlation (r^2^ = 0.95) was found between the publications of terpenoid cancer-related research papers and flavonoid cancer research (Figure 7). On the other hand, terpenoid cancer-related research was negatively correlated with the increments in carotenoids (r^2^ = 0.89). That could explain the importance of the chemical groups, which informs the following target studies regarding anticancer groups’ positive and negative selections.

Recent studies from 2024 to 2025 have significantly advanced our understanding of the anticancer properties of plant-derived secondary metabolites in cancer-related research. These bioactive compounds, including alkaloids [65], flavonoids [66,67], terpenoids [2,4,68], and phenolics [69], exhibit diverse mechanisms of action, such as apoptosis induction, cell cycle arrest, and inhibition of angiogenesis. For instance, Zhou et al. [70] demonstrated that plant secondary metabolites can inhibit cancer by targeting the Epidermal Growth Factor Receptor (EGFR), affecting various signaling pathways like MAPK, VEGF, Ras/Raf, and NF-kβ [71].

A comprehensive review has highlighted the anticancer potential of 144 bioactive compounds derived from plants, emphasizing their effects through pathways such as STAT-3, PI3K/Akt, and Ras/MAP-kinase. Compounds like capsaicin, ouabain, and lycopene have shown efficacy against multiple cancer types via the STAT-3 pathway. Similarly, epigallocatechin gallate and emodin target the JNK protein, while berberine, evodiamine, lycorine, and astragalin affect the PI3K/Akt and Ras/MAP-kinase pathways [72]. In addition, the advancements in plant metabolomics have further elucidated the role of plant-based anticancer drugs [4]. Notable examples include camptothecin derivatives, paclitaxel, and vinca alkaloids, which have been pivotal in cancer therapy. These compounds interact with various molecular targets, exhibiting effects such as antioxidant, anti-inflammatory, antitumor, and anticarcinogenic properties [22].

Specific plant metabolites have also garnered attention for their anticancer properties. For example, Calaf et al. [73] reported that noscapine, an alkaloid, has demonstrated efficacy in treating hematological malignancies. Similarly, paclitaxel, isolated from *Taxus brevifolia*, has selective cancer-fighting properties by inducing the formation of abnormal mitotic spindles, leading to mitotic arrest and apoptosis in cancer cells [74]. These studies collectively underscore the therapeutic promise of SPMs in cancer treatment, highlighting their diverse mechanisms of action and potential for overcoming existing therapeutic challenges.

For the aforementioned reason, Xing et al. [53] reported that SPMs, such as terpenoids, alkaloids, catechins, curcumin, and flavonoids, could be separated into different groups based on their varying regulatory impacts on cancer cell pathways. For instance, these groups have different implications for liver cancer, pancreatic cancer, and gastric cancer, which are common cancer diseases. The catechins group, for example, affects liver cancer by regulating apoptosis and the Hypoxia-Inducible Factor (HIF) signaling pathways. Additionally, they significantly influence tumor necrosis factor-alpha (TNF-α), which could regulate liver cancer invasion and metastasis. For example, a different trend could be observed from curcumin, which regulates the epithelial–mesenchymal transition (EMT) of liver cancer cells and governs the phosphoprotein 53 (P53) gene in gastric cancer cells. Another example is flavonoids, which could regulate pancreatic cancer through their impacts on Ki67 and EMT. Additionally, for hepatocellular carcinoma, their effects stem from regulating AMPK. Along with that, Atanasov et al. [75] noted that SPMs are crucial in drug discovery, particularly for cancer-related diseases.

## 8. Critical Analysis of Plant SPMs’ Pathway Mode of Action

Cancer cells proliferate rapidly and rely heavily on high metabolic activity. In part, natural bioactive phytochemicals may exert their anticancer effects by modulating the nuclear factor kappa B (NF-kB) pathway activity. For example, many SPMs have been reported to have protective and therapeutic properties against various types of cancer. The induction of apoptosis, inhibition of metastasis, and inhibition of proliferation are some of the primary mechanisms by which these molecules act as anticancer agents. Overall, the health-promoting benefits of SPMs have been attributed to the broad range of phytochemicals they contain. It has been reported that these compounds have a synergistic effect, enhancing their apoptotic impact and regulating cancer cell growth [1].

For example, the combination of quercetin and ellagic acid produces a more significant inhibition of cancer cell growth compared to each substance used alone. Li et al. [76] reported that human lung carcinoma cells treated with a natural bioactive leaf extract (200 mg/mL) for 48 h showed a significant decrease in lung cancer cell viability.

## 9. Intervention of Plant SPM Metabolites Based on Their Chemical Structure

### 9.1. Hexacyclic Compounds

Grouping SPMs according to their hexacyclic structure has benefits for illustrating this critical group of antioxidant linkages. Table 4 summarizes the total number of publications for this group of compounds from 2000 to 2023, along with their target genes, pathways, and mode of action. Among the primary hexacyclic bioactive compounds are the quinones derived from *Salvia miltiorrhiza*, which are recognized for their potent anticancer properties [77]. It has been noted that Tanshinones, which are abietane-type norditerpenoid quinones (such as Tanshinone I, Tanshinone IIA, and Dihydrotanshinone I), possess a remarkable ability to regulate the cytochrome of cancer cells. These compounds disrupt mitochondrial membrane potential, increase the Bax/Bcl-2 ratio, and activate caspase-3, all associated with the apoptosis pathway [78,79,80]. Their mechanism of action could be through epigenetically suppressing acetylation of histone H3 associated with cancer genes. Additionally, the zeylenone cyclohexene oxide compound isolated from *Uvaria grandiflora* Roxb decreases the viability, invasion, and metastatic growth of human prostate cells by downregulating the Wnt/β-catenin pathway [81].

Studies have shown that tanshinone IIA prevents HCT116 and HT-29 lung cancer cells from proliferating by lowering TNF-α and IL-6 production in hepatocellular carcinoma cells [82]. Indeed, Chichirau et al. [83] mentioned that two primary mechanisms of cytotoxicity are commonly associated with the quinone mode of action. The first mechanism occurs when a nucleophile reacts with the quinone at the β position to the carbonyl group. This particular reaction can lead to the conjugation of glutathione and subsequent depletion if the nucleophile is glutathione, or it can result in the conjugation of protein thiols, leading to a loss of the cellular protein function. The second mechanism of cytotoxicity involves intracellular redox cycling, a chain reaction that generates superoxide radicals. This initiation step sets off a chain reaction that produces superoxide ions, which can then be converted into hydrogen peroxide through either uncatalyzed or SOD-catalyzed disproportionation. According to Fang et al. [84], tanshinone IIA exhibits a remarkable ability to inhibit the elevation of PU.1, thereby enhancing the production of miR-155 and contributing to its anti-inflammatory and anti-cancer benefits. Additionally, it may increase INF2-related mitochondrial fission by elevating IL-2 levels, which leads to apoptosis in cholangiocarcinoma cells.

**Table 4:** Number of publications, target genes, target pathways, and modes of action for hexacyclic compounds.

SPMs Molecule	PubMed *	Target Genes	Target Pathways	Mode of Action	Reference
Tanshinone IIA	560	c-Myc, STARD13, Nrf2, GCLC, NQO1, P53, and HO-1.	Apoptosis and miR30b-P53-PTPN11/SHP2 pathway.	The suppressive effect on c-Myc gene binding patterns can significantly trigger P53 activation and enhance RNAPII enzyme phosphorylation, resulting in apoptosis. Additionally, it downregulates the miR-125b level while upregulating the target gene STARD13 (StAR-related lipid transfer protein 13).	[77,85]
Tanshinone I	560	Bcl-2, Bid, ITGA2, PPAT, AURKA, VEGF, PI3K, Akt, PRK, JNK, MMP9, ABCG2, AMPKα, PARP, Bax, and Caspase-3.	Akt/Nrf2, SAPK/JNK, PI3K/Akt/mTOR, JAK/STAT3, and ATF-2/ERK kinases.	Disruption of mitochondrial membrane potential (MMP) induces apoptosis in liver cancer cells, inhibits their proliferation, downregulates membrane fluidity, and suppresses the expression of the anti-apoptotic protein Bcl-2.	[86,87,88,89]
Thymoquinone	703	P-Akt, P65, XIAP, Bcl-2, COX-2, VEGF, NF-κB, Bcl-2, XIAPs, Bax, Bid, PARP, GRP78, CHOP, Rac1, and Caspase-3.	Inhibits the growth of cancer cells through the downregulation of PI3K/Akt, STAT3, VEGF, NF-κB, the non-protein sulfhydryl pathway, lactate dehydrogenase, and creatine kinase.	Induces the phosphorylation of extracellular signal-regulated kinase (ERK), MMP, Akt, and cyclic AMP-activated protein kinase-α (AMPKα). Inhibits Akt and AMPKα while inducing the nuclear localization of Nrf2 and the expression of HO-1. Induces the generation of ROS.	[90,91]
Dihydrotanshinone I	69	TNF-α, COX2 (Cyclooxygenase-2), IL-8 in the DOX, and NF-κB.	Activate IKKs (IκB kinases) to induce the inactivation of cytokine expression.	Inactivates NF-κB, which is sequestered in the cytoplasm by phosphorylated IκB (inhibitor of NF-κB) on serine residues.	[92,93]
Zeylenone	12	Bcl-2, Bcl-xl, Bax, and Caspase-3.	Hsp90/Akt/GSK3β apoptosis and necrosis pathway, PI3K/Akt/mTOR, Akt/GSK3β signaling, ERK mitochondrial apoptotic pathway, Fanconi anemia (FA) pathway, and Chk1/P53 pathway.	A 13.2 μM treatment induced a loss of MMP (*p* < 0.01) and ATM/Chk activation in DNA damage-mediated cycle arrest and phosphorylation of Chk and P53. This led to a decline in the anti-apoptotic proteins Bcl-xl and Bcl-2, coupled with an increase in the pro-apoptotic protein Bax, resulting in decreased levels of pro-caspase-3.	[94,95]
Cryptotanshinone	246	IGF1R, MEK1, IRS1, PIK3CA, STAT3, EGFR, ERBB2, mTOR, ERK	STAT3 signaling pathway.	It has a high affinity for binding to STAT3. A dose of 2.5–10 μM decreases the elevated expression of MuRF1 and MAFbx/Atrogin-1 in C2C12 myotubes.	[96,97]

*: PubMed cancer research no. (2000–2023).

In contrast, Li et al. [85] reported that tanshinone is capable of targeting the androgen receptor (AR), STAT3, phosphoinositide 3-kinase/protein kinase B/mammalian target of rapamycin (PI3K/Akt/mTOR), and MAPK, all of which regulate cancer cell proliferation. Additionally, SPMs can downregulate the expression of cyclin D1 and CDK4, which subsequently leads to cell cycle arrest in LNCaP cells [84]. However, Won et al. [98] reported that in AR-silenced LNCaP cells, tanshinone IIA did not affect cell P53 signaling and cyclin of prostate cancer cells. Thus, it is worth noting that different subclasses of tanshinone exhibit varying activities based on the target cancer cells. For instance, Alam et al. [77] reported that the effects of various concentrations of tanshinone I, tanshinone IIA, dihydrotanshinone, and cryptotanshinone on lung cancer cells confirmed that tanshinone IIA displayed superior activity compared to other subclasses through its highly significant influences on G2/M phase by downregulating cyclin A, cyclin B, aurora A, p-cdc and, CDK2 proteins [99].

### 9.2. Phenolics

Phenolic SPMs encompass a variety of hydroxybenzoic and hydroxycinnamic acids, including protocatechuic acid, ferulic acid, and p-coumaric acid. These compounds can be found in nature and are classified as basic 6- or 9-carbon skeletons, namely benzoic acids or cinnamic acids [100]. The categorization of SPMs based on their phenolic structure has the potential to profoundly influence the investigation of their novel anticancer properties, primarily through the functional characteristics of the phenolic rings. This was the reason for the high number of publications in cancer research related to this group of chemicals from 2000 to 2023, based on their unique mode of action (Table 5). For instance, it has been observed that punicalagin, a phenolic compound found in certain plants, exhibits apoptotic activity and exerts inhibitory effects on the proliferation of prostate cancer cells, specifically PC3 cells. These effects are achieved through the upregulation of caspase-3 and caspase-8 expressions, both of which are essential factors in programmed cell death. Moreover, mangiferin, the primary polyphenol in mango peel, has been extensively studied and reported to exhibit a wide range of beneficial effects in various types of cancer, particularly prostate cancer. Its effects include immunomodulatory properties, apoptosis induction, angiogenesis inhibition, and gene expression regulation, as demonstrated in both in vitro and in vivo studies.

Curcumin, for instance, consists of two benzene rings modified by hydroxyl and methoxy groups, with the two benzene rings connected by a seven-carbon ketoenol linker. This molecule has demonstrated anticancer activity against various tumors through specific mechanisms that promote cell apoptosis. Curcumin effectively inhibits the proliferation of cancer cells, such as PC3 and DU145 cells, in a manner contingent upon the dosage and duration of exposure. This inhibition is achieved by the downregulation of the Notch-1 signaling pathway-mediated expression of MT1-MMP and MMP2 in DU145 cells, which consequently hampers the invasion ability of these cells. Wang et al. [101] have demonstrated that curcumin possesses the ability to inhibit the proteins Caspase-3, PARP, and p-MLKL, thereby instigating apoptosis and necroptosis in PC3 cells. Furthermore, its anticancer effect may be mediated through the ERK1/2 and SAPK/JNK signaling pathways, which regulate the expression of P65 and MUC1-C [102].

**Table 5:** Phenolics’ number of publications, target genes, target pathways, and modes of action.

SPMs Molecules	PubMed *	Target Genes	Target Pathways	Mode of Action	Reference
Genistein	3008	CDK1, TERT, TR, EGFR, PDGFR, IR, Abl, Fgr, Fyn, and Src	PI3K/Akt pathway, Cyclin B1	Increase p21 expression, which inhibits HER2 and NF-κB signaling. ZAP-70 expressing cells become activated. It activates caspase-3, inhibits TGF-β-induced EMT, and inhibits NFAT1. It also inhibits FAK expression and enhances the efficacy of EGFR inhibitors. Furthermore, it downregulates NF-κB expression and prevents NF-κB DNA binding.	[103,104]
Protocatechuic	246	PI3K, P-Akt, PKCε, Bax, Bcl-2, caspase-3, P53, and PARP	Apoptosis, Ras/Akt/NF-κB, RhoB, RhoA, PI3K/Akt, Rac1, and Cdc4 pathways	Downregulate MMP-2 and TIMP-2 production. Upregulating the formation of RhoB/PKCε complexes in cancer cells at 25 μM for 8 h significantly reduces Bcl-2 and PARP expression. It induces Bax expression, which is responsible for the intrinsic apoptotic pathway.	[105,106]
Gallic	1530	P21, P53, Mcl-1, Caspase-3, Bcl-2, CD31, VEGF, JNK, GRP78, NF-κB, Nrf2, HO-1, NF-κB, PCNA, FAS, NF-κB, b-Raf, p-MEK, Akt, EGFR-1, VEGF, Bad, MDR1, and PARP	Migration, metastasis, apoptosis, ferroptosis, P53/IL-6/STAT3 pathway, cell cycle arrest, oncogene expression, and M2 macrophage polarization	Reducing anti-apoptotic Bcl-2, nuclear ataxia-telangiectasia mutated (ATM), matrix metalloproteinases (MMPs), tissue inhibitors of metalloproteinases (TIMPs), urokinase plasminogen activator (uPA), and its receptor (uPAR) regulates the activity of hypoxia-inducible factor-1α (HIF-1α).	[12,107,108]
Cinnamic	2534	TGF-β1, iNOS and COX-2, NF-κB, claudin-2, Akt, and ESR1	Vascular endothelial growth factor (VEGF), Bax/Bcl-2, phosphorylation of the P65 subunit and its binding affinity to NFκB, TNF-α protein expression, LPS-mediated pathway, MAPK3	Induces cell cycle arrest at the G0/G1 phase through regulating G1-related protein expression (Cdk4), triggers apoptosis by inhibiting the Akt/Bad pathway, and depolarizes the mitochondrial membrane potential while increasing ROS release.	[109,110,111]
Curcumin	8682	COX-2, NF-κB, Akt, LOX, STAT3, AP1, IL-1, IL-2, Bcl-2, and Bcl-xL, IL-6, EGFR, PDGF, leukemia inhibitory factor (LIF), TNF-α, oncostatin M, MAPKs, ERK1/2, and CNTF	Interferon-γ (IFNγ) pathway, and phosphorylation of the P65 subunit and its binding affinity to NFκB	Increases the production of pro-inflammatory molecules, such as cytokines and ROS. Inhibits phosphorylation by IκB kinase (IKK). Downregulates genes that are anti-apoptotic, mitogenic, and pro-angiogenic.	[57,112]

*: PubMed cancer research no. (2000–2023).

Similarly, catechol, a compound found in certain plants, has been shown to inhibit the proliferation of prostate cancer cells, specifically PC3 cells. This inhibition is achieved through multiple mechanisms, including the generation of reactive oxygen species (ROS), a decrease in mitochondrial membrane potential, DNA damage, activation of caspase-3 and 9 essential enzymes involved in apoptosis, an increase in the Bax/Bcl-2 ratio (pro-apoptotic and anti-apoptotic proteins), and the induction of cell cycle arrest at the G2/M phase. These findings highlight the potential of catechol as a therapeutic agent for cancers. Additionally, the chemical resveratrol can be found in various dietary sources, including *Arachis hypogaea*. It has demonstrated efficacious chemopreventive and chemotherapeutic properties across multiple cancer models.

Additionally, resveratrol (3,5,4-trihydroxy-trans-stilbene) in pomegranate promotes autophagy by suppressing the Wnt/β-catenin signaling pathway. Studies have demonstrated that resveratrol amplifies the oxaliplatin-induced apoptotic effects on liver cancer cells (HepG2), resulting in a synergistic anticancer impact. This compound can lower the ATP levels in HT29 cells, encourage macrophages to produce IL-1β and prevent them from releasing IL-10.

Research has demonstrated that p-coumaric acid exerts an inhibitory effect on producing nitric oxide (NO) and ROS, facilitating anti-inflammatory effects. This action occurs by inhibiting nuclear factor erythroid 2-related factor 2 (Nrf2), highlighting the compound’s potential as an anti-inflammatory agent. Mechanistic studies have revealed that p-coumaric acid exerts its anti-inflammatory action through the suppression of iNOS and p-IκB protein expression, as well as the reduction in NF-κB and IκBα mRNA expression, leading to the inhibition of HCT116 cell proliferation and induction of cell apoptosis. Additionally, these SPMs facilitate the direct release of nitric oxide and reactive oxygen species by macrophages, which target tumor cells, thereby inhibiting their growth and spread. Furthermore, this cytotoxic agent stimulates immune responses, attracting other immune cells’ participation and inducing immune–inflammatory reactions in the tumor microenvironment [113].

### 9.3. Flavonoids

The clustering of flavonoid SPMs in one group could benefit the study of this important group’s anticancer biofunctional mechanisms, based on epidemiological in vitro and in vivo evidence. These compounds form a group of natural phenolic substances that are abundant in various plant organs as secondary metabolites. They play essential roles in many biological processes, including cytotoxicity and anti-inflammatory influences. Flavonoid bioactivity depends on structural substitution patterns in their C6-C3-C6 rings [114]. For instance, capsaicin is a polymethyl flavone belonging to the flavonoid cluster and is obtained from *Capsicum annuum*, also known as Vitex negundo, with anti-inflammatory and anticancer properties. Studies have shown that capsaicin can reduce the carcinogenesis associated with chronic colitis in mice by suppressing NF-κB and MAPK signaling in lipopolysaccharide-stimulated mouse macrophages, thereby inhibiting COX-2 and iNOS expression. Its mode of action involved inhibiting M2 macrophage polarization while promoting M1 phenotypic differentiation of macrophages in the tumor tissues. A similar impact was observed in the vitexin flavonoid compound of the Vitex negundo plant, particularly in oral and other related cancers [115]. The molecule significantly decreased the production of the proinflammatory mediators, TNF-α, IL-1β, and IL-6, compared to the model group. Moreover, recent studies have shown that isoliquiritigenin, a flavonoid derived from licorice, reduces prostaglandin E2 and nitric oxide levels, induces apoptosis, and inhibits the development of aberrant crypt foci [116]. Isoliquiritigenin, a compound within the flavonoid group, is found in herbal medicines such as *Glycyrrhiza uralensis* [117]. It has a similar effect to casticin in decreasing the production of nitric oxide and prostaglandins in cancer cells and lowering the expression of COX-2 and iNOS proteins, ultimately leading to the death of cancer cells [118].

Another critical example is quercetin, a phenolic flavonoid widely found in herbs. Numerous studies have provided evidence of the mode of action for the anti-liver cancer activity of quercetin [119]. For instance, studies have confirmed that quercetin can modulate the PI3K/Akt/mTOR, Wnt/β-catenin, and MAPK/ERK1/2 pathways. This molecule exhibited a significant inhibitory effect on murine mammary cancer cell growth by targeting the Wnt pathway through the upregulation of Dickkopf (DKK) 1, 2, 3, and 4, which are Wnt antagonists [120]. Pretreatment with 300 µM quercetin demonstrated a remarkable ability to induce mitochondrial ROS and significantly downregulate the phosphorylation of Akt, PDK1, Bcl-2, and the levels of the tumor necrosis factor receptor 1 (TNFR1) [121]. Additionally, Kim et al. [58] reported that 20 μM of quercetin inhibited the phosphoinositide 3-kinase (PI3K)/Akt signaling pathway and promote apoptosis in 4T1 murine mammary cancer cells by regulating Wnt signaling activity.

### 9.4. Carotenoids

Carotenoids exhibit a significant capacity to inhibit the growth of cancer cells, both in experimental models conducted in the laboratory and in living organisms [59]. These molecules can influence the activity of various signaling pathways involved in the movement and invasion of cancer cells, as well as the progression of metastasis. Their signaling pathways include crucial regulators of a process called epithelial–mesenchymal transition, which is responsible for transforming stationary epithelial cells into highly motile mesenchymal cells. Additionally, carotenoids have been found to modulate the expression and activity of several regulatory molecules that play a role in cancer cell migration and invasion, such as matrix metalloproteinases (MMPs), tissue inhibitors of metalloproteinases (TIMPs), urokinase plasminogen activator (uPA), and its receptor (uPAR) [122]. Moreover, they regulate the activity of hypoxia-inducible factor-1α (HIF-1α). This protein is involved in the adaptation of cancer cells to low oxygen levels, a common characteristic of solid tumors.

According to Bae et al. [123], the β-carotene molecule can counteract the encouraging impact of M2 macrophages on gastric cancer cells by blocking the expression of the IL-6/STAT3 pathway and M2 macrophage polarization. Notably, 100 µM of β-carotene increases the apoptotic protein P53 and reduces the anti-apoptotic Bcl-2 and nuclear ataxia-telangiectasia mutated (ATM), all of which induce apoptosis in gastric cancer cells. This molecule can lower the expression of TGF-β1, Caspase-3, GSH-Px, TNF-α, and IL-2, thereby preventing the inflammatory response induced by lipopolysaccharide. Similarly, β-Cryptoxanthin, a natural compound found in certain plants, has been shown to inhibit tumor growth. This inhibition is achieved by activating intrinsic and extrinsic apoptotic pathways, which are cellular processes involved in programmed cell death. Furthermore, β-Cryptoxanthin has also been shown to inhibit the p13k/Akt signaling pathway, a key pathway in cell survival and proliferation. These findings suggest that β-Cryptoxanthin may have potential as a therapeutic agent for prostate cancer, which is closely related to β-carotene and other molecules in the same cluster. Altogether, Wu et al. [124] used an in silico molecular docking network to demonstrate the active ingredient of the β-carotene target network by assimilating and analyzing data as a core example for the carotenoids’ functional groups. Consequently, it can enrich the biological anticancer functions and information regulation pathways of potential targets and analyze the anti-inflammatory signal pathways of these grouped molecules.

### 9.5. Alkaloids

Alkaloids, a widely occurring class of significant and effective components in water extracts, are found in various families, including *Berberidaceae* and Solanaceae [125]. One can classify alkaloids into multiple categories, including organic amines, pyrrolidines, pyridines, isoquinolines, indoles, tropanes, imidazoles, quinazolines, purines, steroids, and others. Among these alkaloids, vinblastine, taxol, and homoharringtonine have been extensively utilized in treating various tumors, employing reactive oxygen species (ROS) and ferroptosis [126]. One of the primary active alkaloids in *Sophora flavescens* is sophocarpine, which exhibits several pharmacological effects, including anticancer and anti-inflammatory properties. Zhang et al. [60] reported that sophocarpine can prevent primary mouse macrophage cells from producing TNF-α and IL-6. Furthermore, it can lower the expression of TNF-α and IL-6 mRNA in RAW264.7 cells. In that study, in vivo research showed that sophocarpine had more noticeable benefits in reducing cachexia in CT26 xenograft mice, as well as in lowering blood levels of TNF-α and IL-6. In addition, isoquinoline and hydroxyquinoline alkaloids, which inhibit the high proliferation of many types of cancer cells, exhibit a significant ability to be grouped based on their shared anticancer functional mechanisms [127]. Studies have shown that hydroxyquinoline has an efficient inhibitory effect on TNF production and nuclear factor of activated T-cells, based on its unique quinoline structure, compared to other SPMs [128].

### 9.6. Terpenoids

Terpenoids, similar to alkaloids, such as vinblastine and homoharringtonine, represent a diverse and abundant group of natural compounds found in plants. These compounds have been widely used in the clinical treatment of various tumors, including the induction of reactive oxygen species (ROS) and ferroptosis. The basic construction of their composition is the isoprene group, with variations in the number of isoprene groups. They can be classified into monoterpenes, sesquiterpenes, diterpenes, and triterpenes. Among these, pentacyclic triterpenes have garnered significant attention due to their more pronounced antitumor activities. Tricyclic terpenes can exert antitumor effects through various mechanisms, including direct inhibition of tumor cell proliferation, induction of tumor cell apoptosis, and reversal of drug resistance. These bioactive compounds present significant research prospects in studying nano-delivery systems, necessitating a comprehensive understanding of their molecular mechanisms in cancer. For instance, terpenoids demonstrated better suppressive activity against breast cancer compared to larger molecules, such as polyphenolics. The reason was attributed to the probable superior activity of the small lipophilic isoprene (C_5_H_8_) unit in scavenging reactive oxygen species (ROS), such as singlet oxygen (^1^O_2_) [129].

**Figure 8:**
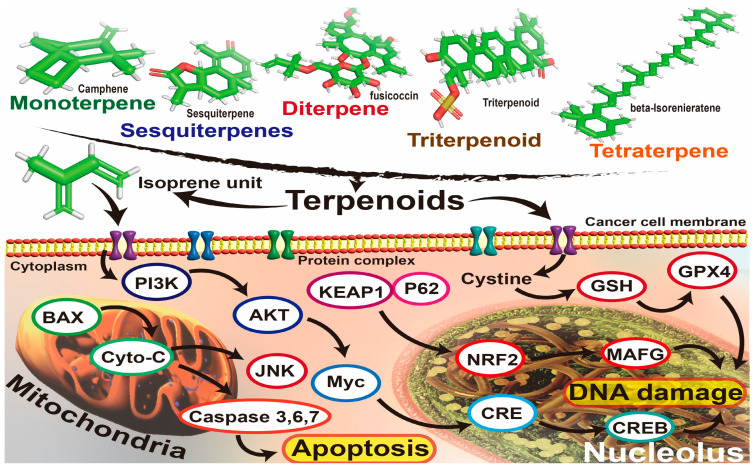
Schematic diagram showing the potential implications of terpenoids on cancer-related pathways and their molecular mechanisms of apoptotic induction [53].

As a notable example of monoterpenes, D-limonene is a monocyclohexene compound consisting of two isoprene units, with a simple molecular formula of C_10_H_16_. This compound is commonly produced from the peels of Citrus sinensis, Citrus limon, and Citrus reticulata [130]. It has a unique feature for cancer therapy, compared to conventional synthetic molecules, which confer advantages and challenges for the drug discovery process [131]. The anti-inflammatory, antiproliferative, and apoptotic effects of d-limonene were observed through its inhibitory impact on the PI3K/Akt cancer-related signaling pathways when combined with Pirfenidone (PFD, a common drug for pulmonary fibrosis). Additionally, Vigushin et al. [132] studied the impact of d-limonene on patients with advanced cancer. In their clinical trials, they found that d-limonene regulated the nuclear transcription factor NF-κB in a dose-dependent manner in aggressive breast cancer cellular lines, affecting pathways involved in cell survival, proliferation, tumorigenesis, and inflammation [133]. Moreover, Wang et al. [134] reported that terpenoids can inhibit the levels of TNF-α, IL-6, and hs-CRP levels in the body. This could be due to the increase in reduced glutathione (GSH) concentration and augmented activity of paraoxonase 1 (PON-1), which collectively play a crucial role by hindering the oxidation of low-density lipoprotein (LDL) (Figure 8).

After treatment with d-limonene at 25–100 mg/kg, rats with lung fibrosis exhibited reduced levels of hydroxyproline (HYP) in lung tissue and decreased serum expression of transforming growth factor beta-1 (TGF-β1) compared to the control group. Additionally, the expression of vascular endothelial growth factor (VEGF) mRNA was downregulated, accompanied by a reduction in the phosphorylation of the P65 subunit of NF-κB, and its binding to NF-κB led to strong inhibition of TNF-α protein expression [135]. A human intervention study confirmed d-limonene’s impact on cancer micro-RNA genes, showing significant downregulation in the expression of cancer genes (such as miR-184, miR-203, miR-373, miR-124, miR-96, and miR-301) compared to the control group.

Another important example is cucurbitacin, a triterpenoid compound derived from the flowers of the Cucurbitaceae family [136]. This compound possesses diverse pharmacological effects, including antitumor and antimetastatic activities. Research has shown that this molecule significantly triggers apoptosis in HCT116 and CT26 cells. Furthermore, cucurbitacin B inhibits the phosphorylation of JAK2 and STAT3 and obstructs their translocation from the cytoplasm to the nucleus. Further experiments have demonstrated that cucurbitacin D reduces the migration of colon cancer cells, as well as endothelial cells, and breast (MCF-7) and central nervous system (SF-268) cancer cell lines [137]. Moreover, by preventing galectin-3 from entering the nucleus, cucurbitacin B dramatically reduces the transcriptional activity of TCF/LEF-1-driven cells. In addition, Lin et al. [138] reported that cucurbitacin B enhances antitumor immune function by promoting the expression of CD4 and CD8 within the tumor microenvironment, significantly restraining tumor growth and metastasis in C57BL/6 and BALB/c colon cancer mouse models [139].

## 10. Emerging Technologies for Detecting Plant SPM Structures and Their Bioactivities

Integrating physical and chemical technologies for the characterization of natural phytochemicals has been enhanced to explore the mechanism of action of these bioactive constituents in biological cells and tissues, thereby maximizing detection efficacy and understanding the spatiotemporal changes in their chemical composition [140]. Additionally, the importance of using microRaman technology for in situ tracking of SPMs inside living plant cells and their bioactivities has drawn the researchers’ attention to studying these compounds’ metabolic pathways and how a biotechnological approach could direct their bioactivities [141].

### 10.1. Real-Time Tracking Technologies for Plants’ SPM Bioactivities onto Cancer Cell Metabolism

**Figure 9:**
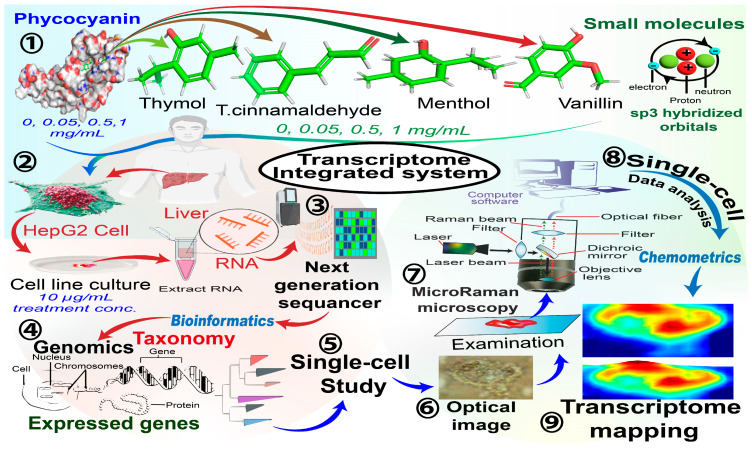
Mechanistic transcriptome protocol for tracking the implications of terpenoids and functional plant protein (Phycocyanin) on single liver cancer cells (HepG2). (1) Phycocyanin molecular structure integration with SPMs (ranging from 0 to 1 mg/mL). (2) The treatment of HepG2 cells under controlled conditions. (3) After the treatment, RNA extraction from the HepG2 cells. (4) Analyzing the extracted RNA using bioinformatics tools, enabling the classification of genes based on their expression profiles. (5) Single-cell RNA sequencing investigation. (6) Optical imaging techniques for the cell morphology. (7) microRaman Microscopy for the biochemical composition of the single-cell. (8) Single-cell Data Analysis. (9) Transcriptome Mapping of specific genes or pathways affected by the small molecules. (Adapted from Gouda et al. [2]; Copyright permission no: 6013401284629).

Increasing concerns about gene annotation quality have raised alarms regarding the need to support the interpretation of obtained results. Such negative impacts have led scientists to employ phenotype methods, such as Western Blot, to demonstrate changes in protein expression. These methods are chemically dependent and cannot detect expression changes in living cells during growth [142]. Therefore, microRaman single-cell tracking technology has significant implications in the current study by mapping chemical changes and modeling the best WNs related to changes in the cellular transcriptome of the membrane and its different organelles [143] (Figure 9).

In that study, the authors overviewed the pheno–genotype complex mechanistic cellular expression for the transcriptomic revolutionary discoveries in single-cell biology [144]. The development of phycocyanin (PC)–isoprene constructs was utilized to modulate the specific activation of hepatic cellular metabolism for microenvironmental spatial detection by RNA-sequencing–micro-spectroscopy [145,146]. This precise and nondestructive approach introduces a complete explanation of cellular level-based phenotype dynamics based on subcellular genomic variation at high resolution [14,147]. Several models were used to verify the stability and repeatability of the fabricated method. Interestingly, PLS-DA, PCA, and k-means clustering models contributed value to this study’s data processing.

Mechanistic studies demonstrated that the capacity of PC to modulate several factors related to cancer cells could be based on its polymerization degree [148]. In addition, the potential cellular redox activity may stem from the increasing reactivity of PC-vanillin (VAN) with oxidizing radicals, which prevents the dissociation of both intramolecular and intermolecular disulfide bonds. This process is necessary for regulating protein activity, which affects several cellular signaling pathways and enzymatic reactions [146]. Additionally, the authors found that the THY group at 1143.19 cm^−1^ was positively correlated with HSP90AB1, as identified as a top-expressed gene in the PLS-DA analysis. Interestingly, it was significantly correlated with acetylneuraminate-9-O-acetyltransferase (CASD1; r^2^ = −0.347; *p*-value = 0.022) and lysophospholipase-2 (LYPLA2; r^2^ = 0.374; *p*-value = 0.009) [2].

Altogether, the help of PC in emulsifying single-structure phytochemicals enhanced the interaction of these small molecules with cancer cell proteins by decreasing their hydrophobicity, thereby facilitating greater interactions with the cell membrane. Indeed, this action was evaluated for its potential to increase the functionality of PC and small SPMs against cancer cell proliferation. Therefore, the changes in the used probes were tracked with the consistency of the highly pure combination of functional PC protein and the different small molecule structures [149].

In contrast, this method defines the potential transcriptomic changes influenced by using bioprobes, where features can be directly associated with specific genes or structures in microRaman high-throughput spectral analyses that may lack clear chemical attributions, making it easier to use prediction models at the single-cell level. Additionally, bioinformatic tools helped unify the top genes from the 10,000 DEGs selected for further inclusion in the microRaman study data analysis [150]. Furthermore, this methodology characterized complex spatiomolecular information from spectroscopy, which still has not achieved high level accuracy with spectroscopic technologies in the case of genomic transcriptional bases. TMicroRaman can preserve single-cell structural information without any destructive preprocesses that may affect the cells [147]. On the other hand, the microRaman technique is still dealing with sizeable spectral data processing. Amazingly, computational models used in the current study, such as variable importance in prediction (VIP) scores, had the advantage of selecting the most significant wavenumbers for prediction. A comprehensive database pipeline could then be obtained for transcriptomic genes using micRaman spectra [2].

### 10.2. Nanoparticle Applications in Spectroscopy and Metal Oxide Sensors for SPM Biomarker Integration Technologies

Metal oxide sensors influence biomarker screening. Stimulated Raman scattering (SRS) represents the most efficient technique, emphasizing the importance of metal oxide sensors. Kubota et al. [151] contributed significantly to the field by developing a confocal Raman microscopic method specifically designed to detect limonene and related bioproducts in cellulose acetate. Their innovative approach employs a deuterium-labeling technique, enhancing the sensitivity and specificity of the analysis. This methodological advancement offered valuable insights into the interactions and behaviors of these compounds within the polymer matrix. In that study, they concluded that Raman band shifts from deuterium labeling could markedly improve the differentiation of limonene in systems with other monoterpenes. Moreover, Zhang et al. [34] visualized the limonene synthesis metabolon within living cells using hyperspectral SRS microscopy. In that study, the colocalization of limonene and limonene synthase was confirmed by co-registered SRS and two-photon fluorescence imaging. Due to its non-destructive and ultrasensitive characteristics, this approach has generated significant attention for the bio-detection of SPM organic compounds. For instance, the potential structure of the limonene has been detected in real-time by using SERS technology. When a molecule’s inherent signals are used, this method creates a “molecular fingerprint” that may be used to recognize a molecule or confirm its existence in a sample. The technique has been discovered to offer several benefits, including high sensitivity, high affinities for molecules, and fingerprint resolution, which may significantly increase the signal strength up to 10 orders of magnitude. Altogether, Kumar et al. [152] reported the limonene chemotaxonomic of *Cipadessa baccifera* and *Xylocarpus granatum* using electrospray ionization quadruple time-of-flight mass spectrometry (ESI-Q-ToF-MS/MS) and collision-induced dissociation (CID) mass spectrometric analysis of fragmented compounds. They reported that MS/MS spectra revealed different fragmentation pathways for limonene.

### 10.3. Nanotechnology in Terms of Particles and Sensors for SPM Biomarker Integration

Another important application is using metal oxide sensors to discriminate between simple volatile derivatives. This technology has become a crucial technique for significantly studying volatile phytochemical discrimination. Combining biomolecules with metallic nanoparticles, such as gold, creates interesting features for developing nanosensors. In our recent study, we developed a novel method for detecting plant cell antioxidants through their single-cell current using a gold nanoprobe [29]. The most straightforward pathway is to use metal nanoparticles, such as gold or aluminum, for the detection process [153]. Meanwhile, the critical dimension of these microelectrodes is small, with nano-diameters that can detect very low concentrations of molecules. As a principal, these sensors’ mechanisms are based on changes in electrical values (such as electric current, resistance, and impedance) triggered by volatile molecules. Nazir et al. [154] developed electrochemical sensors using nanogold (Auphytochemicals) to track limonene concentration, using thiol-capped Auphytochemicals and cyclic voltammetry.

**Figure 10:**
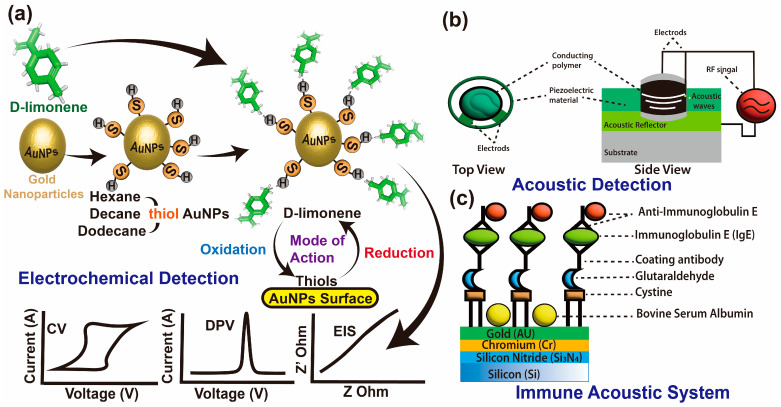
(**a**) Schematic diagram of limonene oxidation at the electrode surface for its electrochemical detection [154]. (**b**) Schematics of quartz crystal microbalance (QCM) acoustic sensor detection (vertical and side views). (**c**) Graphic depicting, in general terms, the immune system’s development in detecting the functional proteins of cancer cells [140] (Open access license).

The sensors’ response to limonene results from complex chemical processes involving the reaction of the receptor–transducer element to oxygen chemisorption (Figure 10a). Therefore, the continuous development of intracellular electrochemical detection, and its relationship with chemical interactions on their surface area, should be further solidified to increase their sensitivities.

### 10.4. Acoustic Sensors for SPM Biomarker Integration Technologies

An emerging detection technique using acoustic-based sensors has demonstrated high sensitivity in measuring the chemical composition of limonene and generating full chemical structure images, which has become one of the hot scientific research areas. In contrast, an acoustic wave sensor typically consists of a piezoelectric substrate (e.g., quartz crystal) coated with sensing material (polymeric film) and two interdigital transducers (one input and one output) commonly used for chemical composition purposes. The acoustic wave propagating through the substrate is called bulk acoustic quartz crystal microbalance (BAW-QCM) (Figure 10b). Wen et al. [155] developed a system based on the quartz crystal microbalance (QCM) acoustic sensors coated with ethyl cellulose (EC), which was designed to detect d-limonene from Australian citrus. Their results mentioned that QCM detected d-limonene at concentrations ranging from 60 mg m^−3^ to 6000 mg m^−3^, with a 0.98 *R*^2^ determination coefficient and a 300 mg m^−3^ limit of detection. In addition, the emerging detection technique using acoustic-based sensors has proven its sensitivity for measuring the chemical composition of limonene, enabling the creation of complete chemical structure images, further validating its relevance as a leading-edge approach in detection technologies [140].

## 11. Integration Between the Emerging Technologies

Special attention has been paid to advanced technologies that have significant potential in facilitating SPM biomarker evaluations [2,29]. Indeed, as noveland fast methods, these techniques can be used to create instant libraries that are compatible with pathways affected by diverse phytochemicals. For instance, an important application of these emerging technologies in the field of cancer pheno–genotype research, in response to SPM treatment, is the interdigitated electrochemical electrode (IDE), which is a highly sensitive metal oxide sensor that can discriminate between molecules released by single cancer cells. This technology demonstrates unique integration for cancer cell-level studies and their prefiltration mechanisms. For instance, the acoustic wave-based biosensor has been used to study the electroacoustic-based mode of action in colon cancer cells. In contrast, the immune acoustic system uses wave sensors that typically consist of a piezoelectric substrate (e.g., quartz crystal) coated with sensing material (polymeric film) and two interdigital transducers (one input and one output), commonly used for analyzing the chemical composition of cancer cell proteins (Figure 10c). Baumgartner et al. [156] discussed the importance of surface acoustic wave-based sensors as noninvasive tracking tools for cancer cell progression. Aptamer-based leaky surface acoustic wave biosensors have demonstrated highly sensitive detection of breast cancer cell metabolism [56]. Notably, the importance of acoustic wave biosensors in chemical phenotyping and discrimination of cancer cells and tissues has been extensively applied in cancer research.

## 12. Limitations of the Current Review

The limitations in the discussed technologies, including the complexities in spectral data, particularly in techniques such as Raman spectroscopy, result in extensive datasets that necessitate sophisticated computational methods, such as multivariate analysis and bioinformatics [6]. While lab-on-a-chip technologies, like QCM, show potential for scalability, they have yet to be widely adopted in industry [155]. Several studies require in vivo validation; for example, the application of d-limonene in clinical trials remains limited [135]. There are experimental shortcomings regarding the bioavailability of substances such as curcumin, which is known for its poor absorption, highlighting the need for optimized nano-delivery systems, such as phycocyanin emulsions. Furthermore, the synergistic effects of combination SPM therapies (e.g., quercetin + ellagic acid) need more adequate exploration for their multi-target approaches [26].

## 13. Conclusions and Future Remarks

The current difficulties facing the SPM technological industry and their viable processing technologies are the main weaknesses in the cycle of proper pathway mechanisms for their wide range of health promotion and treatments. This emphasizes the importance of studying plant cell metabolomics and their released SPMs in response to their functionalities in animal cells. For instance, industrial processes of SPMs could change these molecules’ conformations and consequently change their categorized pathways. Future research should prioritize incorporating AI techniques, such as machine learning, for enhanced spectral analysis. Additionally, employing supercritical CO_2_ for terpenoid extraction is a noteworthy green extraction method. Lastly, a thorough investigation of clinical translation through Phase II/III trials for SPM-related drugs (e.g., cryptotanshinone) is essential. Thus, for producing an effective database related to these compound’s functionalities, every step—from detection inside plant cells using spatiotemporal instant tracking technologies to understanding the exact pathway mechanisms during these different applications, through their extraction processes and evaluation using cancer cells as an important model of the application—should be significantly emphasized. More importantly, addressing the relevant changes in the physicochemical composition of SPMs, along with the reduction of their antioxidant and other bioactivities, could be a solution to overcome a major challenge in this field [130]. Therefore, more suitable and easier-to-manipulate molecular genomic processes should be further investigated using emerging nondestructive techniques for their usage as functional ingredients. The future perspective is to combine sensitive small SPMs with other nano-deliverable molecules to enhance their bioavailability and absorption efficiency after long-term preservation. However, optimizing these new technologies for further large-scale application development plays a vital role in the SPM industry. Additionally, the extraction process should be standardized to provide a greener alternative to commercial chemical and physical methods.

## Figures and Tables

**Table 2:** Real-time tracking and spatial mapping techniques with concentrations and wavenumbers.

Technique	Description	Applications	Concentrations Used	Wavenumbers (cm^−1^)	Reference
**Stable Isotope Probing (Sis)**	It utilizes stable isotopes to track metabolic pathways in real time.	Monitoring the production of bioactive compounds and tracking nutrient use.	^13^C-glucose: 1–10 mM ^15^N-ammonium: 1–5 mM ^2^H_2_O: 30–70% (*v*/*v*)	1150, 1520 (detection via Raman shifts)	[31]
**Hyperspectral SRS**	Combines Raman spectroscopy with hyperspectral imaging for 3D molecular maps.	Visualizing the biosynthesis of metabolites and mapping the distribution of lipids and carotenoids.	Limonene: 10–100 µM Lipids: 50–200 µM Carotenoids: 100–500 µM	- Limonene: 1640 - Lipids: 1440, 1650 - Carotenoids: 1150, 1520	[34]
**Integration of Sis and Raman**	Combines Sis and Raman spectroscopy for real-time metabolic tracking.	Tracking Paramylon Biosynthesis and Monitoring Metabolic Activity in Microalgae	^13^C-glucose: 1–10 mM ^2^H_2_O: 30–70% (*v*/*v*)	- Paramylon: 1150, 1520 - Lipids: 1440, 1650 - Proteins: 1000, 1650	[30,36,38]

## Data Availability

Data will be available upon request.

## Abbreviations

Akt: protein kinase B; CDKIs: cyclin-dependent kinase inhibitors; EGFR: epidermal growth factor receptor; FOLFOX: fluorouracil oxaliplatin; FRP: FTF regulatory protein; GDP: guanosine diphosphate; GTP: guanosine triphosphate; HCFC2: host cell factor C2; IL: interleukin; JAK: Janus kinase; LPR: low-density lipoprotein receptor-related protein; MCM: minichromosome maintenance protein complex; MDM2: mouse double minute 2 homolog; MMP: matrix metalloproteinases; NF-κB: nuclear factor kappa-light-chain-enhancer of activated B cells; PTEN: phosphatase and tensin homolog; RTK: receptor tyrosine kinase; SHOC: leucine-rich repeat protein; SLPs: solid-lipid-particulate system; SPMs: small plant metabolites; SOX: SRY-box transcription factor; STAT: signal transducer and activator of transcription; Thymol: THY; VEGFR: vascular endothelial growth factor receptor; WIF: Wnt inhibitory factor; Vanillin: VAN.
